# Regularities of Structure Formation in 30 mm Rods of Thermoelectric Material during Hot Extrusion

**DOI:** 10.3390/ma14227059

**Published:** 2021-11-21

**Authors:** Mikhail G. Lavrentev, Vladimir T. Bublik, Filipp O. Milovich, Viktoriya P. Panchenko, Yuri N. Parkhomenko, Anatoly I. Prostomolotov, Nataliya Yu. Tabachkova, Nataliya A. Verezub, Mikhail V. Voronov, Ivan Yu. Yarkov

**Affiliations:** 1 Department of Materials Science of Semiconductors and Dielectrics, National University of Science and Technology MISiS, Leninsky Prospekt 4, 119049 Moscow, Russia; lavrentev.mikhail@gmail.com (M.G.L.); bublik.vt@misis.ru (V.T.B.); milovich.fo@misis.ru (F.O.M.); parkhomenko.in@misis.ru (Y.N.P.); m1706006@edu.misis.ru (I.Y.Y.); 2 RMT Ltd., Warshavskoe Shosse 46, 115230 Moscow, Russia; 3 State Scientific-Research and Design Institute of Rare-Metal Industry «Giredmet», Elektrodnaya St. 2, 111524 Moscow, Russia; ViPPanchenko@giredmet.ru (V.P.P.); MVVoronov@giredmet.ru (M.V.V.); 4 Ishlinsky Institute for Problems in Mechanics, Russian Academy of Sciences, 119526 Moscow, Russia; aprosto@inbox.ru (A.I.P.); verezub@ipmnet.ru (N.A.V.); 5 Prokhorov General Physics Institute RAS, Vavilova St. 38, 119991 Moscow, Russia

**Keywords:** bismuth telluride, hot extrusion, thermoelectric material, texture, mathematical simulation, thermoelectric figure of merit

## Abstract

In this study, Ingots of (Bi, Sb)_2_Te_3_ thermoelectric material with p-type conductivity have been obtained by hot extrusion. The main regularities of hot extrusion of 30 mm rods have been analyzed with the aid of a mathematical simulation on the basis of the joint use of elastic-plastic body approximations. The phase composition, texture and microstructure of the (Bi, Sb)_2_Te_3_ solid solutions have been studied using X-ray diffraction and scanning electron microscopy. The thermoelectric properties have been studied using the Harman method. We show that extrusion through a 30 mm diameter die produces a homogeneous strain. The extruded specimens exhibit a fine-grained structure and a clear axial texture in which the cleavage planes are parallel to the extrusion axis. The quantity of defects in the grains of the (Bi, Sb)_2_Te_3_ thermoelectric material decreases with an increase in the extrusion rate. An increase in the extrusion temperature leads to a decrease in the Seebeck coefficient and an increase in the electrical conductivity. The specimens extruded at 450 °C and a 0.5 mm/min extrusion rate have the highest thermoelectric figure of merit (Z = 3.2 × 10^−3^ K^−1^).

## 1. Introduction

Bismuth and antimony chalcogenide based solid solutions are the main thermoelectric materials used by the manufacturers of thermoelectric cooling and generator modules [1,2]. The materials of thermoelectric module cells work under severe thermal conditions and loads. The temperature gradients produced in these materials during operation may induce large thermal stresses which can ultimately cause destruction of the material and module cell failure. For this reason, the technology of these materials is the key aspect in the fabrication of thermoelectric devices, its importance being greater when it comes to the fabrication of miniaturized cooling systems for microelectronics, optoelectronics, and laser devices where the thermoelectric material quality and reliability requirements are extremely stringent [3].

Bismuth and antimony chalcogenides have a rhombohedral structure with the $R\bar{3}m$ symmetry group, and their physical properties are anisotropic [4]. Their parameters, such as their electrical conductivity and heat conductivity in the direction of the third order symmetry axis and in directions perpendicular to this axis, may differ by several times (hence the anisotropy of the thermoelectric figure of merit) [5].

The most widely used technologies of Bi_2_Te_3_ based materials are zone melting [6,7,8], spark plasma sintering [9,10,11,12], and hot extrusion [13,14,15]. The preferable technology is hot extrusion for which plastic strain produces a predominant grain orientation (i.e., a texture) in the polycrystalline material and, as a result, the final ingots exhibit good mechanical and thermoelectric properties [16,17,18].

Hot extrusion technology is a well-known and practically important process for synthesizing bismuth telluride-based thermoelectric materials. However, the need to increase the process output and the product quality gives impetus to permanent improvement of both the geometry of the dies used and the process parameters (e.g., die extrusion rate) [19,20].

The aim of this work is to obtain homogeneous 30 mm diameter ingots of (Bi, Sb)_2_Te_3_ solid solutions by hot extrusion and to study the effect of process parameters on the structure and thermoelectric properties of the material.

## 2. Materials and Methods

The p-type conductivity (Bi, Sb)_2_Te_3_ thermoelectric material was synthesized by direct smelting of the raw components taken in the stoichiometric ratio in quartz ampoules at 750 °C. The synthesis duration was 2 h. The raw components for solid solution synthesis were as follows: 99.999 wt.% pure tellurium, 99.999 wt.% pure antimony, 99.999 wt.% pure bismuth. The synthesized material was crushed in an XS-10 blade mill (Hebei, China). The as-milled powder size was <500 μm. The thermoelectric material powder was preliminary compacted in an IP2500 M auto hydraulic press (Armavir, Russia) with a specific pressure force of 3 g/cm^2^. The hot extrusion process was carried out in the following mode: temperature 400 ÷ 500 °C, extrusion rate 0.1 ÷ 0.5 mm/min, and extrusion coefficient 10.

The structure of the samples was studied by X-ray diffraction and scanning electron microscopy (SEM). The samples for X-ray diffraction studies were disks with a diameter of 30 mm and a thickness of 3 mm, which were cut from different parts of the extruded rod perpendicular to the axis of extrusion. After cutting, a broken layer is formed on the surface of the samples, the structure of which differs from the structure in the volume of the material. Before the study of the structure, the samples were etched in a solution of HBr and K_2_Cr_2_O_7_ (1:1) for 5 min to remove the broken layer.

The phase composition of the specimens was studied by X-ray diffraction on a Bruker D8 instrument (Karlsruhe, Germany) with CuKα incident radiation. The scan time per step was fixed to 1 s with the step size of 0.02° in the 2θ range of 15°–105°. The fine structure (crystallite size and microdeformations) was assessed by comparing the broadening of the first and second order diffraction peaks (HKL and 2H2K2L) using the reference profile. The reference was an as-annealed Bi_0.4_Se_1.6_Te_3_ solid solution powder. The texture was characterized by plotting reciprocal pole figures based on the X-ray diffraction patterns taken for sections perpendicular to the extrusion axis (so as to evaluate the probability of coincidence between poles for different planes and the extrusion axis). The statistical weights of the poles were calculated with normalization with respect to the calculated reflection intensities. The morphology of bulk specimens was characterized under a scanning electron microscope (JSM-6480LV, JEOL, Tokyo, Japan). The grain size was determined using an SEM of the cleaved surfaces. The samples were chipped at room temperature. As chalcogenide grains cleave preliminarily along the cleavage planes of the chalcogenide structure, the cleaved surfaces show the grain structure of the material. The sizes of the cleaved structural features were assessed using the intercept method.

The thermoelectric parameters (electrical conductivity, Seebeck coefficient, thermal conductivity, and thermoelectric figure of merit) of the materials were measured using the Harman method at room temperature on 2.5 × 2.5 × 4.0 mm^3^ samples cut from the ingots parallel to the extrusion axis [21,22].

## 3. Results

The extrusion technology has a number of specific features which may deleteriously affect the electrophysical properties of extruded branches. The large strain developed in the extruded material triggers processes which may destabilize the properties of the branches and impair their parameters.

The conditions of extrusion process (die shape, strain temperature and rate, amount of strain and workpiece structure) affect the final structure and properties of the extruded material. One effective method of studying the effect of plastic molding parameters on the structural parameters of the thermoelectric material is to develop a mathematical simulation of the extrusion process.

A mathematical simulation of extrusion process can deliver information on a number of process parameters that cannot be studied experimentally such as stress, strain, and strain rate fields both at the final stage and in evolution during rod extrusion. Mathematical simulation provides the possibility of compare the evolution of these parameters with the development of the structure and to identify the process stages which prove to be the most critical ones for the structure formation in extruded rods. Simulation of virtual extrusion processes under various boundary conditions (in particular for different die designs given the same rod diameter) provides an opportunity to significantly reduce the effort for the fabrication of expensive equipment. The regularities of the hot extrusion process for 30 mm diameter rods of the (Bi, Sb)_2_Te_3_ thermoelectric material were analyzed by the joint use of elastic-plastic body approximations.

### 3.1. Mathematical Simulation of Extrusion Process

**Figure 1:**
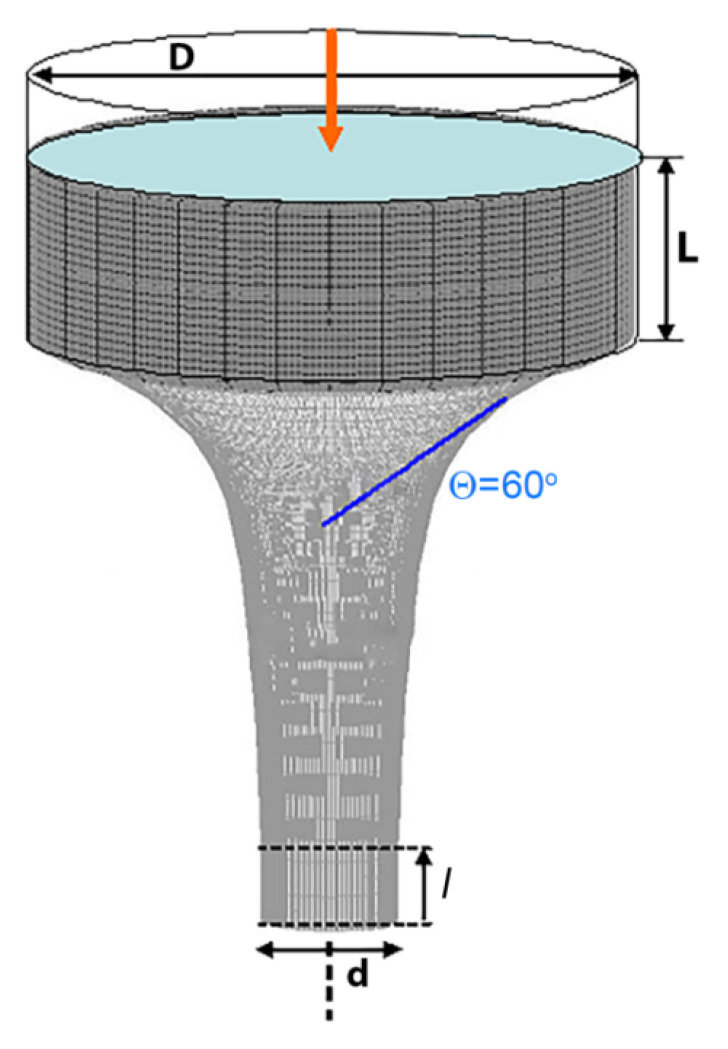
The scheme of hot extrusion process.

The scheme of the hot extrusion process for mathematical simulation is shown in Figure 1. In accordance with the schematic presented above, the geometrical parameters of the hot extrusion plant were set up as follows: D = 85 mm is the diameter, L = 26 mm is the length of the workpiece, and θ = 60 deg is the die rounding angle; l = 10 mm is the length and d = 30 mm is the diameter of the cylindrical section at the die output. Also, a process rate of V = 0.1 mm/s was set up. The D^2^/d^2^ parameter describes the hot extrusion process efficiency and is referred to as the extrusion ratio.

**Figure 2:**
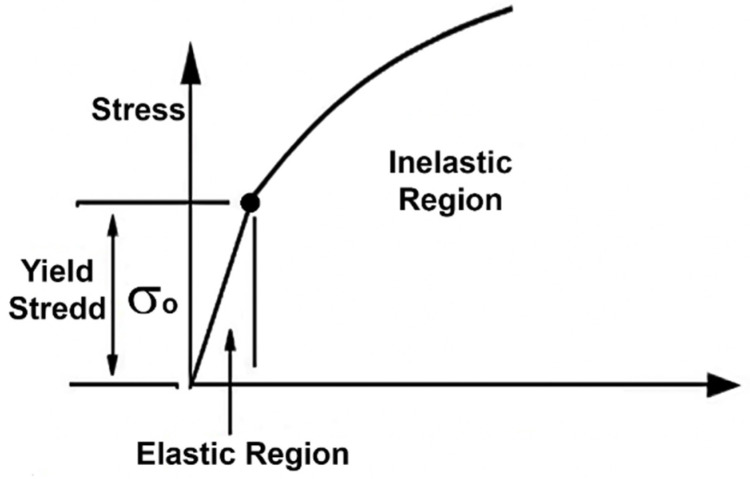
Stress vs. strain graph for elastic-plastic approximation.

The physical and mechanical parameters of the process were chosen in accordance with earlier work [19]: E = 40 GPa is Young’s modulus, ν = 0.3 is Poisson’s ratio, and σ_o_ = 102 MPa is the elastic to plastic transition threshold stress. The elastic to plastic transition is illustrated in the stress vs. strain graph in Figure 2. As reported earlier [19], the friction coefficient between the workpiece and the die is 0.04. The model takes the friction into account but the calculations ignored it due to the process uses graphite lining for workpiece slipping.

#### 3.1.1. Methodical Approach to Calculation of Elastic-Plastic Strains during Hot Extrusion

The methods used in this work are based on a solid-state approach and joint use of elastic and plastic body approximations in accordance with the fundamentals of the elasticity and plasticity theory [23]. Thermal stresses can be ignored for hot extrusion processes. A detailed justification of the choice of this approximation was reported earlier [24]. Data on an alternative approach based on the mechanics of rheological liquid media were provided elsewhere [25] for cold extrusion of high plasticity materials. We will now consider the fundamentals of the elastic-plastic approximation used in this work.

The dependence between stresses and strains for an elastic isotropic body are as follows: if σ_1_, σ_2_, σ_3_ and ε_1_, ε_2_, ε_3_ are the main stresses and strains then, considering them as being related to the main axes, one can establish the following relationship between them:

σ_1_ = (λ + 2G) ε_1_ + λ ε_2_ + λ ε_3_, σ_2_ = λ ε_1_ + (λ + 2G) ε_2_ + λ ε_3_, σ_3_ = λ ε_1_ + λ ε_2_ + (λ + 2G) ε_3_ (1)

where λ is the Lamé coefficient and G is the shear modulus. Young’s modulus E determines the relationship between the stress and the relative elongation during tension:

E = σ_1_/ ε_1_ = G(3λ + 2G)/(λ + G) (2)

Poisson’s ratio determines the relationship between the transverse strain and the longitudinal strain:

ν = λ/2(λ + G)(3)

The strain continuity equation is as follows:

∂^2^ε_x_/∂y^2^ + ∂^2^ε_y_/∂x^2^ = ∂γ_xy_/∂x∂y (4)

The stress balance equations are as follows:

∂σ_x_/∂x + ∂τ_yx_/∂y + ∂τ_zx_/∂z + ρX = 0    ∂τ_xy_/∂x + ∂σ_y_/∂y + ∂τ_zy_/∂z + ρY = 0 ∂τ_xz_/∂x + ∂τ_yz_/∂y + ∂σ_z_/∂z + ρZ = 0 (5)

The invariants are as follows:

I_1_ = σ_x_ + σ_y_ + σ_z_, I_2_ = − (σ_y_σ_z_ + σ_z_σ_x_ + σ_x_σ_y_) + τ^2^_yx_ + τ^2^_zx_ + τ^2^_xy_ (6)

When the plasticity conditions are considered, the hydrostatic pressure is excluded from the common set of equations and the remainder is referred to as the deviator of stresses and it is accepted that the latter parameter determines the onset of the plastic transition and is the only tool to for expressing the plasticity condition. The average stress is determined as follows:

s = (σ_x_, σ_y_, σ_z_)/3 = (σ_1_, σ_2_, σ_3_)/3 (7)

which is an invariant value. The stress deviator is determined by the components s_x_, s_y_, s_z_, s_yz_, s_zx_, s_xy_ in accordance with the following equations:

s_x_ = σ_x_ − s, s_y_ = σ_y_ − s, s_z_ = σ_z_ − s, s_yz_ = τ_yz_, s_zx_ = τ_zx_, s_xy_ = τ_xy_ (8)

The strain components determine the average strain:

e = (ε_x_, ε_y_, ε_z_)/3 = (ε_1_, ε_2_, ε_3_)/3 (9)

Then the strain deviator components e_x_, e_y_, e_z_, e_yz_, e_zx_, e_xy_ are determined by the following expressions:

e_x_ = ε_x_ − e, e_y_ = ε_y_ − e, e_z_ = ε_z_ − e, e_yz_ = γ_yz_, e_zx_ = γ_zx_, e_xy_ = γ_xy_ (10)

The mathematical notation of the plasticity condition follows from the Tresca condition [23] of the greatest tangent stresses, which states that plastic strain onsets at the point where the greatest tangent stresses reach the value σ_o_/2 which is the constant of a material. Since the greatest tangent stresses are (σ_1_ − σ_3_)/2, Trask’s condition is written as follows:

(σ_1_ − σ_3_) = (s_1_ − s_3_) = σ_o_ (11)

This condition determines the similar values of the yield stress σ_o_ for uniaxial tension and compression. One of the requirements imposed on the plasticity condition is its invariance with respect to the coordination axes. Moreover, it is assumed that the average normal stress does not affect the plasticity and that, therefore, the plasticity condition can only be expressed in stress deviator components. This results in the necessity of studying the invariance of the stress deviator. The stress invariants J_1_, J_2_, J_3_ are introduced formally and by analogy with the stress invariants I_1_, I_2_, I_3_:

J_1_ = s_x_ + s_y_ + s_z_, J_2_ = − (s_y_s_z_ + s_z_s_x_+ s_x_s_y_) + s^2^_yx_ + s^2^_zx_ + s^2^_xy_ (12)

The simplest case corresponds to J_2,_ which is considered constant and referred to as the von Mises condition:

2J_2_ = s^2^_1_ + s^2^_2_ + s^2^_3_ = 2σ_o_^2^/3 or (σ_2_ − σ_3_)^2^ + (σ_3_ − σ_1_)^2^ + (σ_1_ − σ_2_)^2^ = 2σ_o_^2^/3 (13)

where σ_o_ is the constant of the material.

In accordance with the von Mises condition [23], flow occurs when the shape change elastic strain energy reaches the characteristic value for the material in question.

Ignoring elastic strains we will consider plastic flow for a two-dimensional (2D) case. The principal stress in the z axis direction will then be ½(σ_1_ + σ_2_). In this case the von Mises plasticity condition takes on as follows: σ_1_ − σ_2_ = 2k, where k= σ_o_/3^1/2^. For the stress components along the xy axes, this expression follows:

¼(σ_x_ − σ_y_)^2^ + ∂τ_xy_^2^ = k^2^ (14)

For a 2D case the stress balance Equation (5) have the following form:

∂σ_x_/∂x + ∂τ_xy_/∂y = 0 ∂τ_xy_/∂x + ∂σ_y_/∂y = 0 (15)

Repeatedly differentiating Equation (15) and subtracting it, we obtain taking into account Equation (11) the following:

∂^2^τ_xy_/∂x^2^ − ∂^2^τ_xy_/∂y^2^ = ± 2∂^2^/∂x∂x√ (k^2^ − τ_xy_^2^) (16)

Equation (16) is solved relative to τ_xy_. From the calculated stresses one can find the strain rate:

s_x_ = 2φε’_x_, s_y_ = 2φε’_y_, s_xy_ = 2φγ’_xy_ (17)

The tangential friction stress is related to the stress normal to the surface by the following relationship: σ_t_ = −μσ_n_ t, or for acting forces: f_t_ = −μf_n_ t, where f_t_ is the tangential friction force, f_n_ is the normal force, σ_t_ is the tangential friction stress, σ_n_ is the normal stress, μ is the friction coefficient, and t is the tangential vector in the velocity direction.

#### 3.1.2. Results of Mathematical Simulation of Extrusion Process

The extrusion process was simulated using the Crystmo/Marc finite elements simulation complex [26]. The mathematical model developed allowed us to carry out a virtual simulation of extrusion process which resulted in the extrusion of cylindrical specimens 20 and 30 mm in diameter. During the calculation, the computational (Lagrange’s) mesh and the specimen shape changed in time at sequential steps of the extrusion process, suggesting that the specimen output from the die starts as early as in 150 s.

For cylindrical specimen 20 mm in diameter the main zones of the stress-strain specimen state have been analyzed in [27], which determine its strength (the high compression zone) and quality (the structure formation zone and the potential longitudinal cracking zone) for the stage of specimen output from the die.

**Figure 3:**
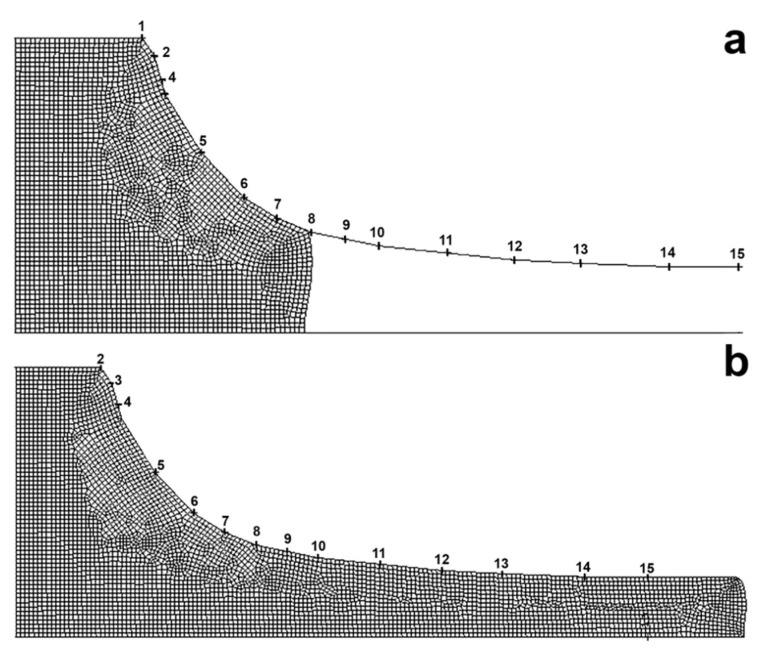
Lagrange mesh and specimen shape vs. time t, s: (**a**) 90, (**b**) 150.

For cylindrical specimens 20 mm in diameter, Figure 3 shows the evolution of the computational (Lagrange) mesh and the specimen shape in time at sequential steps of the extrusion process, suggesting that the specimen output from the die starts as early as 140 s.

**Figure 4:**
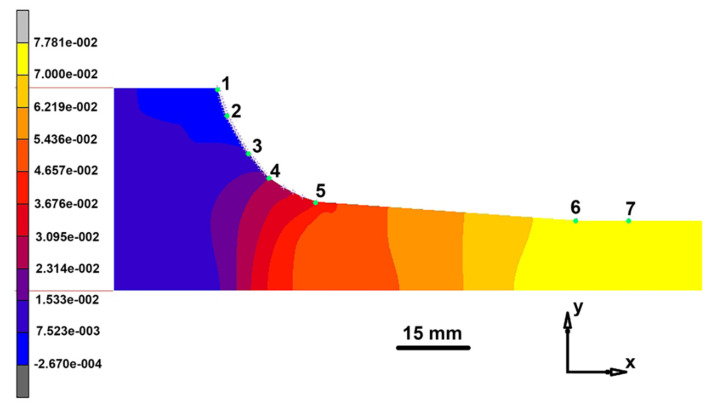
Distribution of plastic flow velocity V during extrusion of 30 mm rod at time 360 s from process start. Values for color isocontours correspond to mass flow velocities V × 0.1 mm/s.

For cylindrical specimen 30 mm in diameter the distributions of the plastic flow velocity V isocontours suggest that at an early process stage (t = 90 s), the flow velocity is higher at the die wall. This is accounted for by the greater contribution of material pressing from the sides (Zone 1) to the center where the compression degree is the highest. However, at the stage of specimen output from the die (t = 360 s) the radial flow profile changes so that the flow velocity in the center becomes greater than near the die wall (Figure 4).

The key parameter for analysis of the stress-strain state of specimens is the time corresponding to the start of specimen output from the die. The absolute mass transport velocity in the conical section of the die (marks 6 and 7) and at the die output is 0.78 mm/s. Near the die surface (marks 1 and 2) the extruded material undergoes counter flow, its zone being expanded in the course of extrusion until the end of the process.

**Figure 5:**
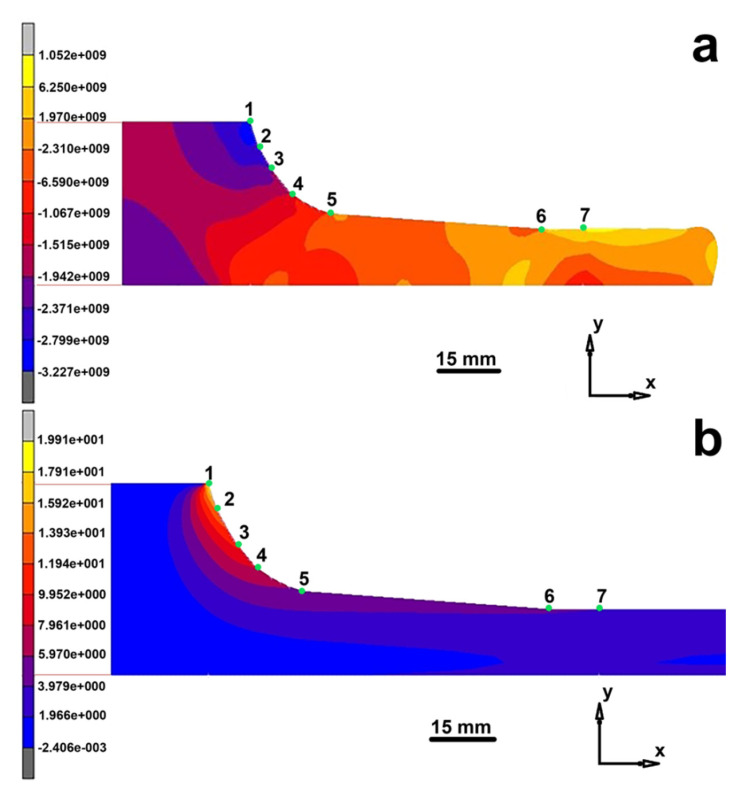
Distribution of main maximum stresses ×10^7^ MPa (**a**) and total maximum strains for extrusion of 30 mm diameter rod (**b**).

In article [27], similar data corresponding to Figure 4 and Figure 5 are cited for 20 mm rod extrusion process.

Figure 5 shows the distributions of the principal maximum stresses and total maximum strains for extrusion of a 30 mm diameter rod.

The stress distribution shown in Figure 5 suggests that at the die throat between side marks 1 and 4 the stress is negative, varying from −280 to −27 MPa which corresponds to a reduction of the tensile stress with an increase in the distance from the press position. Downstream of mark 4 the stress sign changes and the corresponding compression stress reaches approximately 75 MPa. Below marks 6–7 (at the cylindrical die section) and in the free part of the specimen, the radial stress inhomogeneity spans from 67 MPa compression (at the die wall) to 70 MPa tension (in the specimen center). The overall radial stress scatter in the specimen at the die output is about 70% of the respective scatter for extrusion of a 20 mm diameter rod. The strain distribution suggests that the greatest strain (7.0 and 14.0) is developed at the corner point of the die (Figure 5b, marks 1–2). Between marks 2 and 5 the strain decreases to 3.7. Below marks 6–7 and in the free part of the specimen, the strain decreases to 1. This section exhibits radial strain scatter corresponding to the respective changes in the stress state of this specimen section. The comparison of two variants of the die (20 and 30 mm in diameter) shows that more homogeneous strain is developed during a hot extrusion through 30 mm die.

### 3.2. Structural Study of Extruded Rod

**Figure 6:**
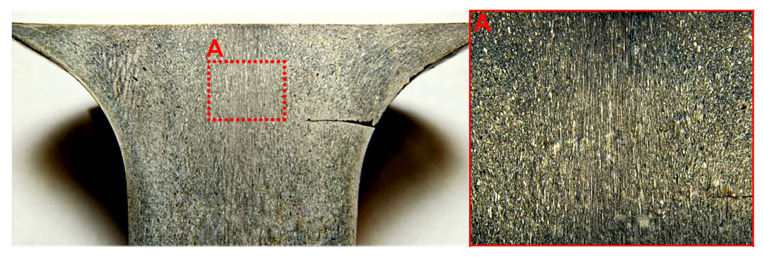
Macrostructure image of the initial section of the extruded rod.

Structural study of 30 mm diameter extruded rods of (Bi, Sb)_2_Te_3_ solid solution showed that the initial press workpiece has a texture in which the (0001) cleavage planes are perpendicular to the strain direction. A strain texture starts to form in the center of the transition zone. Thereafter, the strain texture develops, the predominant grain orientation being such that the (0001) cleavage planes are parallel to the extrusion axis. The microstructure image presented in Figure 6 shows elongated grains or grain agglomerations along the extrusion axis. The extruded rod has a channel in the center in which the flow of material is far more intense as compared with the rest of the initial section of the extruded rod. This strain pattern can be accounted for by opposite pressing of the material by the outer layers.

**Figure 7:**
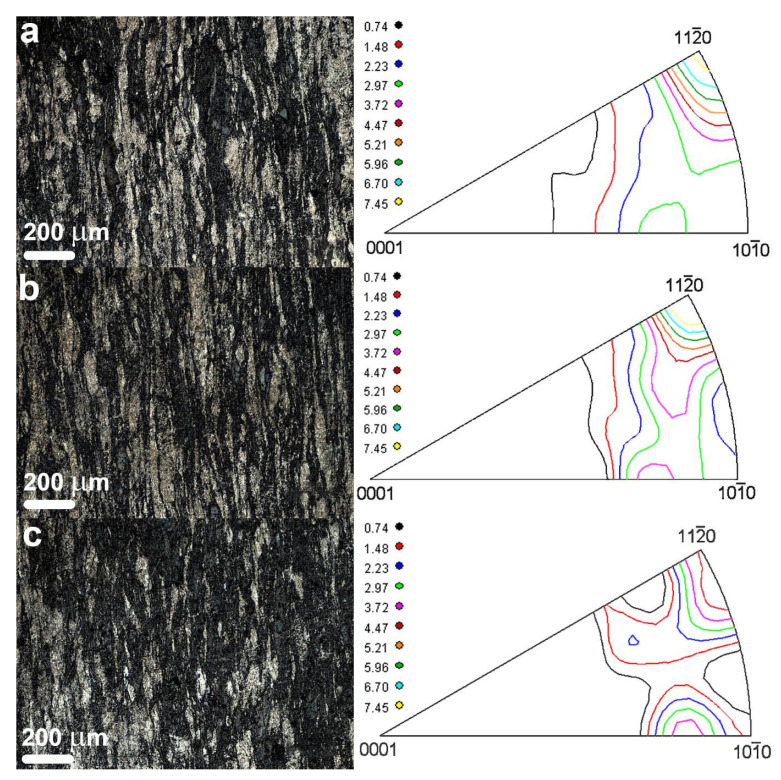
Evolution of rod texture and microstructure along extrusion axis: (**a**) 1 cm from die beginning, (**b**) 3 cm from die beginning, and (**c**) 5 cm from die beginning.

Figure 7 shows the microstructure and inverse pole figures of wafers cut out from the working part of the rod perpendicularly to the extrusion axis. The microstructure image clearly resolves grains or grain agglomerations with close orientations elongated in the extrusion direction. The elongation and orientations of the grains reflect the plastic flow pattern and its radial homogeneity. The texture of the extruded rod is almost similar in the longitudinal direction, the predominant grain orientation being with the cleavage planes parallel to the extrusion axis.

**Figure 8:**
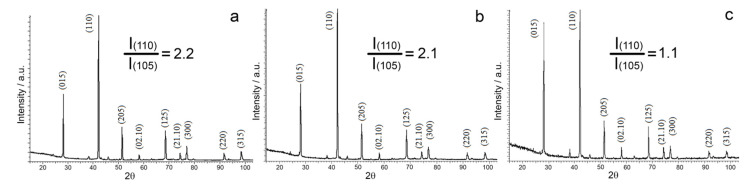
Evolution of diffraction peak intensity as a function of extrusion temperature: (**a**) 430 °C, (**b**) 450 °C, and (**c**) 470 °C.

Figure 8 shows diffraction patterns for wafers cut out from the middle part of the extruded rod for different extrusion temperatures.

Phase analysis data suggest that all of the test materials are single phase. The diffraction patterns exhibit only the (Bi, Sb)_2_Te_3_ solid solution reflections. However, the diffraction peak intensity ratio varies depending on extrusion temperature. For the purposes of illustration, Figure 8 shows intensity ratio between the (110) line for which the cleavage planes are parallel to the extrusion axis and the most intense line for the textureless material (105). With an increase in the extrusion temperature, the texture of the material degrades. As the extrusion temperature grows from 430 to 450 °C, there is a slight change in the intensity ratio of the diffraction peaks, whereas the quantity of the grains whose cleavage planes are parallel to the texture axis remains almost the same at these temperatures. The texture degradation becomes considerable as the extrusion temperature increases to 470 °C. The texture of the material does not change noticeably depending on extrusion rate. An increase in the extrusion rate leads to a decrease in the quantity of defects in the grains. Table 1 summarizes characteristics of the fine structure of the material, i.e., sizes of coherent scattering regions (CSR) and microstrain.

Fine structure characterization (determination of coherent scattering region sizes and microstrain) of the test material showed that the microstrain degree decreases with an increase in the extrusion rate. The average size of coherent scattering regions depends on the extrusion rate (albeit slightly).

**Figure 9:**
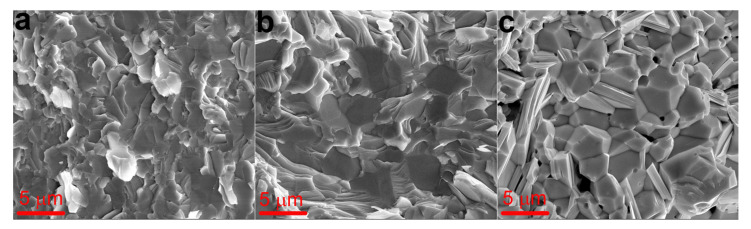
Cleave surface structure images of (Bi Sb)_2_Te_3_ solid solutions extruded at different temperatures: (**a**) 430 °C, (**b**) 450 °C, and (**c**) 470 °C.

**Figure 10:**
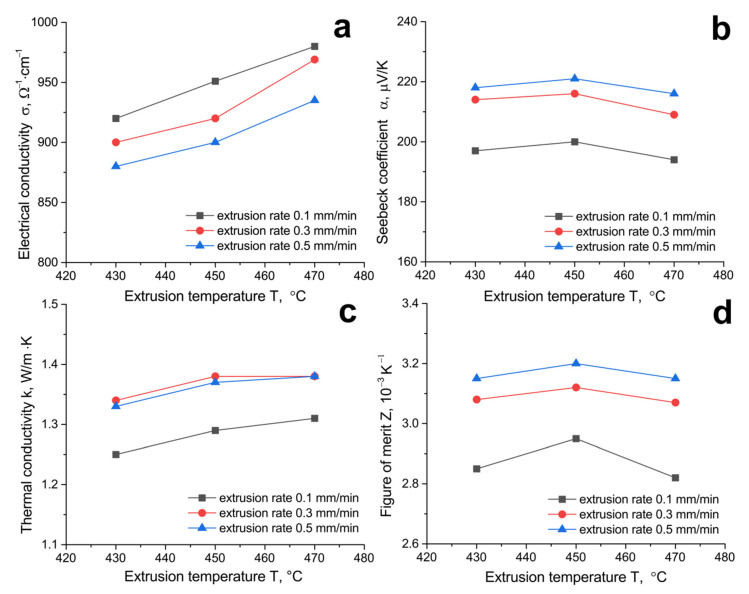
(**a**) Electrical conductivity, (**b**) Seebeck coefficient, (**c**) thermal conductivity, and (**d**) figure of merit as a function of extrusion temperature.

Figure 9 shows images of the cleave surface structure for solid solutions obtained at different extrusion rates. Since bismuth and antimony chalcogenide based solid solutions are cleaved mainly along their cleavage planes, their cleave surface images exhibit textures reflecting the grain structure of the material. Specimens extruded at different temperatures have almost similar sizes of structural features (about 1–5 μm). Only the cleave surface image of the specimen extruded at 470 °C shows pores (Figure 10c). These pores can be seen both along the grain boundaries and inside the grains. Pore formation at this extrusion temperature can be accounted for by coagulation of point defects and/or changes in stoichiometry due to tellurium evaporation.

### 3.3. Thermoelectric Properties of Extruded (Bi Sb)_2_Te_3_ Solid Solutions Depending on Extrusion Temperature and Rate

Figure 10 shows electrical conductivity, Seebeck coefficient, thermal conductivity, and thermoelectric figure of merit as a function of extrusion temperature for different extrusion rates.

As can be seen from the data presented in Figure 10, an increase in the extrusion temperature leads to an increase in the electrical conductivity of the solid solutions. At each experimental extrusion temperature, the electrical conductivity of the solid solutions decreases with an increase in the extrusion rate. The Seebeck coefficient increases slightly with an increase in the extrusion temperature from 430 to 450 °C and decreases noticeably with an increase in the extrusion temperature to 470 °C. The dependence of thermal conductivity on the extrusion temperature correlates well with the electrical conductivity curves. The minimum value of thermal conductivity has a sample obtained at an extrusion rate of 0.1 mm/min. This is explained by the fact that at a low value of the extrusion rate, the compaction process proceeds with the formation of many structural defects. The thermal conductivity of the samples obtained at an extrusion rate of 0.3 and 0.5 mm/min practically does not differ at comparable extrusion temperatures. At comparable extrusion temperatures, the Seebeck coefficient increases with an increase in the extrusion rate. The thermoelectric figure of merit changes nonmonotonically with an increase in the extrusion temperature and has a clear peak at a 450 °C extrusion temperature. Similarly, the Seebeck coefficient changes in Figure 10b. An increase in the extrusion rate leads to an increase in the thermoelectric figure of merit. The specimens extruded at 450 °C with a 0.5 mm/min extrusion rate have the highest thermoelectric figure of merit (Z = 3.2 × 10^−3^ K^−1^).

When the extrusion temperature changes from a temperature of 430 °C to 450 °C, the values of electrical conductivity and the Seebeck coefficient simultaneously increase, and the study of the fine structure shows a decrease in the value of microdeformations at a temperature of 450 °C. Such a change in the electrophysical parameters is probably due to a decrease in grain defects and an increase in the mobility of the main charge carriers. Comparison between the data on the structure and properties of the material suggests that primary recrystallization onsets at 470 °C and leads to structure degradation. Furthermore, boundary migration during primary recrystallization changes the carrier concentration. On the one hand, deformed defects that produce acceptor levels are annealed and, on the other hand, point defects are generated during high-angle boundary migration in the course of recrystallization. In our opinion, the acceptor levels are associated with defect formation during plastic strain due to the intersection of dislocations migrating in different slip planes. Since the main type of defects in the bismuth telluride based solid solutions are tellurium vacancies and cation atoms in antisite positions, the thermal impact during extrusion may cause bismuth atoms to substitute tellurium ones in the antisite positions. Both of these mechanisms lead to the formation of acceptor centers.

The hypothesis of the onset of primary recrystallization in the p-type conductivity material at 470 °C is confirmed by electrophysical data since the electrical conductivity increases and the Seebeck coefficient decreases at this temperature. This change of the electrophysical parameters is caused by an increase in the carrier concentration which is plausible since the defect generation is the most intense at the recrystallization center formation temperature.

The data on the structure and thermoelectric properties reported herein suggest that the optimum extrusion temperature for the (Bi, Sb)_2_Te_3_ solid solutions is 450 °C. At this extrusion temperature, the materials do not contain pores and have a fine-grained structure with a clear axial texture for which the cleavage planes of the grains are oriented along the extrusion axis.

## 4. Conclusions

30 mm diameter ingots of the (Bi, Sb)_2_Te_3_ thermoelectric material were produced by hot extrusion. Mathematical simulation showed that extrusion through a 30 mm diameter die produces a homogeneous strain. Variations of the process temperature revealed regularities in the structure formation and allowed us to choose the optimum extrusion mode for the selected temperature range. We show that p-type conductivity (Bi, Sb)_2_Te_3_ solid solutions with the best properties are obtained at a 450 °C extrusion temperature. At this extrusion temperature, the strain texture is retained and pores do not form. We show that the quantity of defects in the grains of the (Bi, Sb)_2_Te_3_ solid solutions decreases with an increase in the extrusion rate. The specimens extruded at 450 °C with a 0.5 mm/min extrusion rate have the highest thermoelectric figure of merit (Z = 3.2 × 10^−3^ K^−1^).

## Figures and Tables

**Table 1:** CSR size and microstrain in (Bi, Sb)_2_Te_3_ solid solutions as a function of extrusion rate.

Extrusion Rate, mm/min	CSR Size, nm	Microstrain, %
0.1	120	0.16
0.3	115	0.12
0.5	125	0.08

## Data Availability

The study did not report any data.
